# “Makes you proud to be black eh?”: Reflections on meaningful Indigenous research participation

**DOI:** 10.1186/1475-9276-11-40

**Published:** 2012-08-08

**Authors:** Jenny Kelly, Sherry Saggers, Kylie Taylor, Glenn Pearce, Peter Massey, Jennifer Bull, Travis Odo, John Thomas, Rosita Billycan, Jenni Judd, Susan Reilly, Shayne Ahboo

**Affiliations:** 1 School of Public Health, Tropical Medicine and Rehabilitation Sciences, James Cook University, Townsville, QLD, 4811, Australia; 2 National Drug Research Institute, Curtin University, Perth, Australia; 3 Hunter New England Health Service, Tamworth, Australia; 4 Research Centre for Clinical and Community Practice Innovation, Griffith University, Brisbane, Australia; 5 Mamu Health Service Limited, Innisfail, Australia; 6 Kimberley Aboriginal Medical Services Council, Bidyadanga, Australia

**Keywords:** Indigenous, Participatory action research, Aboriginal, Torres Strait Islander, Participation

## Abstract

**Introduction:**

This article outlines the meaningful participation of eight Aboriginal and Torres Strait Islander community members employed as community researchers investigating the impact of pandemic influenza in rural and remote Indigenous communities in Australia. Aboriginal and Torres Strait Islander participation is now a requirement of health research involving Aboriginal and Torres Strait Islander communities. There is a growing literature on the different approaches to such involvement. Fundamental to this literature is an acknowledgement that Indigenous communities are no longer prepared to be research objects for external, mostly non-Indigenous researchers, and demand a role in decisions about what is researched and how it will be researched. In this paper, we describe the protracted process for site identification and recruitment and training of community researchers. We focus on the backgrounds of the Indigenous researchers and their motivations for involvement, and the strengths and challenges posed by Indigenous people researching in their own communities. Throughout the paper our concern is to document how genuine participation and the building of research capacity can occur.

**Discussion:**

A key feature of the research was the employment, training and strengthening the capacity of local Aboriginal and Torres Strait Islander community members in the role of community researchers. A series of training workshops were conducted in northern Australia and focussed on qualitative research methods, including data collection, data analysis and writing. The Indigenous researchers collected the community-based data, and worked in partnership with experienced academic researchers in the analysis and compilation of community reports. Parts of those community reports, as well as additional information supplied by the community researchers, forms the basis of this article. As the demand increases for involvement of Indigenous community members as researchers, focus needs to be paid to what constitutes meaningful participation. If active participation in all aspects of the research process is intended, this necessitates close attention to the knowledge and skills required for this to occur at every stage. Building research capacity means not simply equipping local people to undertake research on a particular project, but to have the knowledge and skills to undertake research in other areas.

**Conclusions:**

There are considerable benefits for Indigenous people researching in their own communities. Most important for the community researchers on this project was the sense that they were doing important health work, not just conducting research. Given the persistent gaps between Indigenous and non-Indigenous health, this is perhaps one of the most important contributions of this type of research. Whilst research outcomes are undoubtedly important, in many cases the process used is of greater importance.

## Introduction

Aboriginal and Torres Strait Islander participation is now a requirement of health research involving Australian Aboriginal or Torres Strait Islander communities [1]. There is a growing literature on the history of and different approaches to such involvement [2-5]. Fundamental to this literature is an acknowledgement that Indigenous communities are no longer prepared to be research objects for external, mostly non-Indigenous researchers, and demand a role in decisions about what is researched and how it will be researched [2]. A search of Indigenous health research reveals a wide range of participation levels by Aboriginal and Torres Strait Islander people [3,6] and we are not suggesting that there is only one way in which authentic participation can be achieved. Indigenous communities may decide that while they want to maintain overall control of a particular project, they do not require local community members to be active researchers on the project. However, if people do want to be actively involved as researchers, this requires close attention to research training and capacity building. Given the relatively lower levels of literacy and formal education among some Indigenous Australians, providing quality research training can be challenging. In this research project, the Indigenous community researchers collected all the community-based data, and worked in partnership with experienced academic researchers in the analysis and compilation of community reports. Parts of those community reports, as well as additional information supplied by the community researchers, form the basis of this article.

This story started with a discussion between some of the team’s non-Indigenous researchers, and community members from Aboriginal communities in a rural area of New South Wales (NSW), Australia about community responses to government plans to control influenza. Aboriginal people told us about their memories of intrusive government surveillance and control measures used on them in the past. These memories had a negative impact on their responses to the current pandemic government policy. These conversations with Aboriginal community members meant that community consultation occurred early in the research process and was indeed the impetus for applying for research funding. We set out to learn from rural and remote community members how the Australian National Action Plan for Human Pandemic Influenza needed to be modified to be appropriate and feasible for Indigenous communities [7].

Under the guidance of the Hunter New England Health Aboriginal Health Partnership (the Partnership), the process of developing a shared understanding of the threat of pandemic influenza for Aboriginal and Torres Strait Islander peoples became the basis for a national research project. The Partnership is a committee comprising government and non-government health services that advise on health planning for Aboriginal people in the Hunter New England area of NSW.

Together with our colleagues from different organisations across Australia we received funding from the National Health and Medical Research Council to study community responses to pandemic influenza in rural and remote Aboriginal and Torres Strait Islander communities. The initial research team comprised university academics including a senior Aboriginal researcher, and senior health professionals employed in both government and community-controlled health organisations. We received approval from the Human Research Ethics Committees in Hunter New England (NSW), the NSW Aboriginal Health Medical Research Council, James Cook University in Queensland and the Western Australian Aboriginal Health Information and Ethics Committee before we started the research.

Details of the broader research project are published elsewhere [8]. The research reported here explores the much used but frequently under-described concepts of participation and capacity building. As with most similar Indigenous research projects, our initial proposal identified Indigenous participation and capacity building as important objectives; however, it was only after we had reported on the primary research aims and objectives around community responses to pandemic influenza that we started to question in more depth how we had addressed these issues. We wanted to better understand how the research process assisted the Indigenous individuals involved to engage with the research, how they reflected upon their participation and the impact, if any, on their future plans. Data was gathered both informally and formally, through unstructured conversations during workshops and field work, and in more focused telephone and email interviews in which the researchers responded to questions about their preparation for and experiences of the research process. Although departing from academic conventions, all the community researchers requested that their reflections be published under their real names. In keeping with the principles of Participatory Action Research (PAR) and not privileging Western knowledge over Indigenous knowledge [4], we have retained the community researchers’ verbatim quotes and used their own names.

### Identification of community sites

The Hunter New England Health Aboriginal Health Partnership, together with the partner organisations involved, supported a PAR approach to explore the research issues, as they believed this best met their requirements for authentic participation. PAR is not simply a research tool, but seeks to bring about positive change in communities through the equal and collaborative involvement of those affected by the issue concerned in a research cycle of planning, action, reflection and action [9].

A starting point for engagement with each community site was an existing relationship. Long standing personal and professional relationships existed between various members of the wider research team and each Indigenous community and Indigenous organisation and these were of vital importance to the success of the project, as relationships are the first step in the research journey [10].

In NSW, Tamworth and Inverell were identified by the Hunter New England Health Aboriginal Health Partnership as appropriate sites for the community research. The Partnership initiated and provided oversight to the research project. James Cook University and Curtin University have long standing relationships with the Kimberley Aboriginal Medical Services Council (KAMSC) in Broome, Western Australia (WA). KAMSC is a regional Aboriginal Community Controlled Health Service that provides a voice for a number of member community controlled health services across the remote Kimberley region of Western Australia. In addition to its support and advocacy role, KAMSC also provides comprehensive primary health care services to three remote communities in the region, including the community of Bidyadanga. It was through these relationships with KAMSC that Bidyadanga was identified as an appropriate site for the WA research.

Existing relationships with the Palm Island Aboriginal Shire Council resulted in the island being selected as a site for the pandemic research. The Mayor of Palm Island contacted an Aboriginal Chief Investigator and requested that the island be included in the research, following an adverse pregnancy outcome in a woman infected with Swine Flu.

In north Queensland, existing community links with an Aboriginal Chief Investigator were the starting point for engagement with a local Aboriginal community controlled health service in the Innisfail region. Through this cultural and familial link, two local Indigenous community members employed at the health service were seconded to the research project. These young people had excellent links with their community and knowledge of community health issues in their local area. Adequate research funding meant we could pay the health service to backfill their positions for the duration of the project. This co-operative arrangement benefitted all the parties concerned. In the Torres Strait, links with a non-Indigenous academic instigated the negotiations and agreement for local community involvement. Table 1 provides details of sites, researchers and their backgrounds, recruitment methods, support provided and subsequent roles.

**Table 1:** Community researchers and sites, recruitment methods, support and subsequent roles

**Sites**	**Researchers**	**Background**	**Recruitment**	**Support**	**Subsequent roles**
Tamworth & Inverell NSW	1M & 1F	EHO* & AHW*	Operational roles in health service	CI on site	Employed by health service.
Palm Island QLD	1F	Community member	Known to Indigenous CI	Research Fellow Townsville, QLD	Community researcher
Innisfail QLD	1M & 1F	AHW & Receptionist	AMS staff nominated	Research Fellow Townsville, QLD	Student & not in paid workforce
Torres Strait QLD	1F	Administration	Advertised locally	Two Researchers Townsville, QLD	Not in paid workforce
Bidyadanga WA	1M & 1F	Community members	Community presentation & EOI called	Researchers from Broome, WA & Townsville, QLD & CI Perth, WA	Employed by health service & AWH training.

*EHO – Environmental Health Officer, *AHW – Aboriginal Health Worker.

### Recruitment of community researchers

In keeping with the guidelines for ethical research in Australian Indigenous studies, we recognised the diversity and uniqueness of Aboriginal and Torres Strait Islander peoples and communities [11] and consequently the process used to recruit community based researchers differed from site to site.

In WA, an Aboriginal health researcher employed at the KAMSC, Broome was actively involved in the recruitment, interviewing, training and ongoing support and mentoring of two remote community-based Aboriginal researchers. The research team placed greater emphasis on community knowledge over formal education and thus the usual recruitment strategies needed to be adapted to meet this need. Rather than advertising a formal position and asking potential applicants to submit an application addressing the selection criteria, two health staff, including an Aboriginal researcher from KAMSC and a non-Indigenous researcher from Townsville visited Bidyadanga and held an open community meeting explaining the project and calling for expressions of interest for the research worker positions. Some three weeks after our visit, two local community members were interviewed and subsequently appointed to the part-time community based researcher positions.

Informal networks and existing links with community members in the Torres Strait assisted in the recruitment of an appropriately qualified community researcher on Thursday Island. A well known and locally respected Torres Strait Islander was employed as a cultural advisor/mentor to the research team to advise and guide the research team in matters related to engagement with the Torres Strait. His cultural and community knowledge identified appropriate people and protocols for respectful engagement to occur. A part-time community researcher position was advertised throughout the Torres Strait and a local woman was employed in the role. Due to the vast distances between the James Cook University, Townsville (where the project was based) and the Torres Strait (approximately 1083 kilometres/673 miles via air) additional funding was required to enable weekly air travel and accommodation for two non-Indigenous research team workers to provide her with weekly support and mentoring.

In NSW, two Tamworth based Aboriginal community members employed by the state government regional health service were responsible for recruitment and interviewing of community members in the Hunter New England region of NSW. Prior to becoming community researchers, these two community members had operational roles within the health service and importantly had extensive knowledge of the issues of their local communities. These health service employees were well supported in their community research role by the guidance and mentorship of one of the Chief Investigators, a non-Indigenous public health professional employed by the health service.

All community based researchers had varied knowledge, skill sets and different educational backgrounds, ranging from no formal post school education to postgraduate public health studies. Where possible, two community members, male and female, were employed at each site and supported to work in their new roles as community researchers. This was not possible in the smaller sites of Palm Island and the Torres Strait, and thus a sole worker was employed. In total, eight Indigenous community members were recruited. Of these, seven were Aboriginal and one, a Torres Strait Islander. All community researchers were of different ages (20s-50s), sex, backgrounds and stage of career with a diversity of lived experiences. Prior to working on the research projects two were employed in a state health service (NSW), another three were not in the paid workforce (WA & Palm Island), and two were employed in an Aboriginal community-controlled health service in north Queensland. In the Torres Strait, a young woman employed outside the health sector was recruited and supported in the role of community researcher by two non-Indigenous researchers and guided by a Torres Strait Islander cultural advisor. Although the community members were novice researchers, they were respected members of the communities in which they lived and worked, with rich understandings of local, social and cultural norms.

### Research training workshops

An overarching aim of our research was to strengthen research capacity within Aboriginal and Torres Strait Islander communities. We successfully budgeted for a comprehensive training process for the research team, including the community researchers, comprising generic and person-centred training. The total budget for the three year project was $1,485,250 (AUD) and apart from salaries for the community researchers and a project manager, the majority of this was spent on research training, related travel and accommodation expenses. A total of nine, two-day generic training workshops (18 days in total) were conducted from 2009 to 2011 across four of the sites in northern Australia. These training workshops focussed on qualitative research methods, including data collection, data analysis and writing. The NSW researchers joined the wider research team at the training in northern Australia which provided the additional benefit of enabling the community researchers from the other sites to meet and support each other in their roles as community researchers. In the remote sites of the Torres Strait and Bidyadanda, the participants were limited to the local community researchers and the research fellows/mentors.

Participants at the larger training workshops included Chief and Associate Investigators, community researchers and project staff. These workshops were conducted at key points of the research journey, ‘just in time’ [12] for it to be relevant and meaningful for conducting research in the field. Each workshop comprised a theoretical component which introduced participants to qualitative research theory and methods, and a practical component in which data collection, analysis and writing techniques were modelled and then practised. This approach to training was in keeping with the principles of adult education which enables students to ‘learn by doing’ [13]. For instance, during the introductory data collection workshop community researchers practised interviewing in a supportive environment prior to interviewing ‘real’ participants in their own communities. They learned how to take brief but accurate interview and focus group notes, having to read these back to their interviewees and the group. Workshop participants also learned the importance of reflection, to identify the strengths and weaknesses of their data collection and how to improve their research skills.

As data collection progressed, analysis workshops provided time for community researchers to work with senior researchers on their own research data before presenting it to the whole group for discussion. At the writing workshops community and academic reports were drafted collectively by the whole team, so community researchers could experience the way in which this type of knowledge is produced and disseminated. This sharing of ideas, experiences and knowledge improved the quality of our work and highlighted the reciprocal capacity strengthening among Indigenous and non-Indigenous researchers. The bringing together of the team enabled ‘two-way’ learning to occur in a supportive learning environment [14]. While senior, non-Indigenous researchers taught research theory and practice, and individually mentored community researchers, the community researchers explained the meaning of the data they had collected and together the team wrote accessible community reports that tried to do justice to the rich data.

Along with these generic workshops the team also provided person-centred training which acknowledged the different literacy levels, knowledge, experience and confidence of the community researchers. This included adapting much of the generic workshop material to suit the needs of the remote community researchers. One of the research mentors, an Aboriginal woman from Western Australia (WA), worked with two community researchers and two non-Indigenous researchers/mentors to adapt the material’s language and examples so that it was more meaningful for the community researchers. This process was facilitated by a skills audit of the community researchers in which they identified their areas of strength and those that required further development. The extra time taken to complete this activity was vital as it enabled us to focus on the researchers’ learning needs, rather than applying a ‘one size fits all’ approach. Additional time and space were allocated to allow the community researchers to conduct mock interviews away from the wider research team, under the guidance of a research mentor. These mock interviews rotated the roles of interviewer, note taker and interviewee. Time was also built into the sessions for reflection and discussion.

Participation in these generic and person-centred workshops and the collaborative nature of the field work meant that all of the research team were able to get to know one another and start to feel more comfortable about raising difficult questions such as “how reliable is this data?” and “how would I do this differently next time?” As our relationships developed we were able to talk more openly about the meaning of research participation and capacity building. In this process the motivations of the researchers and the strengths and challenges of becoming a community researcher were revealed as important.

### The desire to make a difference

A key feature of the research was the employment, training and strengthening the capacity of local Aboriginal and Torres Strait Islander community members as community researchers. Despite the breadth of diversity and life experiences amongst the community researchers there were shared motivational factors for wanting to work as researchers in their own communities. Some of these motivations included the desire to make a difference to Indigenous health.

As Kylie, a community researcher from NSW explained:

"*Growing up I always wanted to do work where I was helping people and making a difference. I didn’t really have my heart set on anything in particular, but knew I wanted to work with people who are disadvantaged or marginalised. My mother Annie was an Aboriginal Health Worker for 15 years and I saw things that she was doing that could make a difference some of the time to some of the people, but to see changes that are sustainable and have long term benefits to more people, you need to change not just the grass roots stuff, but also systems and infrastructure.*"

And similarly, her colleague, Glenn, an Environmental Health Officer (EHO) told us:

"*I was looking for something that would get me closer to community. This research meant that I could have those yarns with people. From my work experience, understanding the housing and environment issues really helped me to see pandemics’ direct impact on the household and the way people live. I saw an opportunity to get involved in research to change the impact of pandemics on Aboriginal communities.*"

For two community researchers living in a remote community in WA, their reasons for wanting to be involved initially reflected a more pragmatic approach. Rosita explained:

"*Well at first I just wanted a job and to do something different with my life instead of staying at home sitting around and doing nothing.*"

Her colleague, John, spoke about the difficulty associated with taking on a new and unfamiliar role:

"*It was difficult coming out of being unemployed and stepping into a researcher’s role because I had to adapt myself into the workforce and start to learn new things.*"

However, as he reflected on his experience he was able to identify benefits both for himself and his community:

"*This project offered me a lot of career options and helped getting me back in the workforce. I was interested in this research project because it was about swine flu. I’d like to see more health awareness projects* [in my community]."

Upon completion of the research project, two WA based community researchers progressed to full time employment with a community controlled health service and have commenced Aboriginal Health Worker Training. Another Queensland community researcher has enrolled in a university-based undergraduate nursing degree program. The two NSW community researchers continue to work in their public health and researcher roles with the state government health service. Two Queensland female community researchers are currently out of the workforce and another from Palm Island is currently involved in other community based research projects.

### The highs and the lows of becoming a community researcher

For all of the community researchers, the opportunity to participate in the research project was a new experience. This new experience brought with it a degree of trepidation for some of the researchers. The difficulties and challenges involved in stepping out of a familiar role and into an unknown environment cannot be underestimated. Travis, a male health worker from north Queensland explained:

"*When we attended the first workshop we were both terrified because we were stepping into a new environment where there were different people we didn’t know. We were so comfortable being in an environment we were familiar with* [the community controlled health service] *it just sort of suffocated both of us. To be really honest I was scared and I felt very sick that first day. I remember when we first walked into the conference room shortly before beginning and I felt very nervous. It didn’t help that I was second in line for doing the little introduction of myself. I can remember everyone after us introducing themselves as Doctors, Nurses and people that had this type of PhD and that type of degree, it was very intimidating. I really wanted to get up and leave and I am very glad that I didn’t because then I wouldn’t be a part of the team that I am proud to be in today.*"

Similarly, his colleague, Jennifer, a female receptionist, employed in the same health service explained:

"*The very first day I was a nervous wreck; it was quite scary because apart from Travis I really didn’t know anyone. I felt very intimidated being in a room full of well educated people coming from all different backgrounds. Throughout the workshop, it was difficult to take things in because it was all new to me and the terminology was a little difficult for me grasp. I wanted to get up and leave, but I’m glad I made the choice to continue. Looking back now, I am proud of us and the work that we have achieved in such a short amount of time.*"

Historically, research with Indigenous peoples has been a colonising practice which placed them as passive objects of curiosity whereby scientists across the world came and examined their bodies, cultures and societies to describe how different they were from the ‘civilised’ world [2]. Aboriginal and Torres Strait Islander people are acutely aware of the practice of researchers taking information from community members without giving anything back. Kylie explained:

"*I used to think that research was bad and it’s still considered a dirty word to many Aboriginal people. Too much research in the past was done ‘on’ Aboriginal people that didn’t benefit people at all. Usually the only benefits went to the non-Aboriginal researchers and academics. Aboriginal people received very little in return for their time, knowledge and valuable contribution to the research.*"

Likewise, Glenn told us:

"*Mum tells the story; she can remember when a linguist came and recorded language on a tape recorder and then the payment for all the years was a couple of dresses. That was the only payment for the wealth of knowledge.*"

For Indigenous community members conducting research in their own communities presents both challenges and rewards. As an Indigenous researcher, a community member has a unique position as an ‘insider’ which assumes a level of ‘taken-for-granted knowledge.’ ‘Taken-for-granted knowledge’ is a quality that Aboriginal and Torres Strait Islander researchers bring to the wider research team. This knowledge meant that the community researchers often felt awkward when they were required to explain concepts to the participants that were known and understood by both the participant and the community researcher.

Kylie informed us:

"*I didn’t feel shame about being involved in research but I felt a bit stupid sometimes when I had to ask Aboriginal people questions when I already knew the answer because of a shared experience. But asking the question was important because even though I had a shared understanding and knew the answer most of the time it was still vital to get the information in peoples’ own words, unedited, unchanged and most importantly – analysed and interpreted exactly the way****they****meant it to be.*"

She elaborated:

"*There is a lot of cultural knowledge that you grow up knowing and it all plays a part in who you become. All that knowledge is just known as it is part of growing up black. Growing up strong in culture, being surrounded by your family and mob all the time, you know your boundaries, limitations, and you know your place. So trying to make sense of the research in that community context, and making the spiritual, cultural and ‘unwritten’ knowledge that more believable to white people was hard to do.*"

The community researchers identified many positive aspects of their research involvement. Some of them found the PAR model to be an ideal way of giving back to community members immediately. As Aboriginal and Torres Strait Islander community researchers, they were able to provide information about pandemic influenza that was useful and appropriate for the participants:

"*When you are interviewing people you get a lot of questions not just answers. One of the highlights is that we had a lot of the knowledge to be able to answer the questions at the time of the interview. This was really important as it was a way of giving back straight away, a way of being able to provide immediate action or advice.*"

Glenn shared these sentiments and commented:

"*Having all the Aboriginal Health Workers involved and connected helped to ground the work, especially the action part of the research. This research was community owned; the information that was developed, the community took ownership and it became theirs. That action part is so important.*"

The Indigenous community researchers also acted as cultural brokers and mentors to the non-Indigenous research team throughout the research process drawing upon their knowledge and lived experiences of two different world views [6]. The non-Indigenous research team members benefitted from learning from the Aboriginal and Torres Strait Islander researchers through the challenging of their assumptions and approaches to research. This collaborative process highlighted the reciprocal capacity strengthening of both Indigenous and non-Indigenous researchers.

## Discussion

The involvement of Indigenous peoples in all aspects of the research agenda is widely understood as a necessary and important aspect of a successful research project [5,15]. As the demand increases for involvement of Indigenous community members as researchers, focus needs to be paid to what constitutes meaningful participation. If active participation in all aspects of the research process is intended, this necessitates close attention to the knowledge and skills required for this to occur at every stage. Indigenous participation in the identification of the research topic was not problematic at all, as the NSW Aboriginal Health Partnership raised pandemic influenza as the topic they wanted researched. They were also clear that they wanted a participatory research approach which would train local community members to carry out the research. Other research has demonstrated the strength of these approaches in Indigenous communities [16,17].

Research training needs to take account of the various backgrounds of the researchers and the motivations they have for wanting to be involved in research. This requires adequate resourcing to adapt materials, provide sufficiently intensive training, and most importantly, on-going supervision and mentoring throughout the research process. We were fortunate to have the funds, time and appropriately qualified and experienced people to provide one-on-one support for significant periods of the research.

Building research capacity means not simply equipping local people to undertake research on a particular project, but to have the knowledge and skills to undertake research in other areas. It should also provide people with a critical understanding of the difference between empowering and disempowering research [18,19]. Authentic research participation brings considerable challenges to Indigenous people. As Travis and Jennifer so eloquently describe, it requires a level of confidence to start regarding themselves as an integral team member. As Kylie illustrated, they also need to unpack ‘taken-for-granted’ community knowledge about how illness is experienced, knowing their interviewees sometimes thought they were stupid for asking questions whose answers were self-evident [2].

Despite the initial fear associated with taking on unfamiliar research roles, there are also considerable benefits for Indigenous people involved in researching in their own communities. Most important for the community researchers on this project was the sense that they were doing meaningful public health work, not just conducting research. Given the persistent gaps between Indigenous and non-Indigenous health, this is perhaps one of the most important contributions of this type of research.

## Conclusion

This paper provides an insight into what constitutes meaningful Indigenous research participation as identified by Indigenous community researchers employed on a large Australian research project. Our research highlighted the complexity of working on a large project with a multidisciplinary team of Indigenous and non-Indigenous researchers located in three states of Australia with a range of backgrounds, skills and career development. Despite the differences in the research team, the shared commitment to social justice approaches to Indigenous health enabled reciprocal capacity strengthening among Indigenous and non-Indigenous researchers. The research plan was based on the PAR cycle of planning, action, reflection and action [9]. Whilst research outcomes are undoubtedly important, in many cases the process used is of greater importance [2]. As Travis remarked after observing an interview by one of his Indigenous community researcher colleagues: “makes you proud to be black, eh?”

## Competing interests

The authors declare that they have no competing interests.

## Authors’ contributions

JK designed, led and drafted the paper. SS provided valuable intellectual input, guidance, mentorship and reviewed several drafts. PM contributed to the content and reviewing and editing of the manuscript. KT, GP, JB, TO, JT, RB provided content for the primary focus of the paper. JJ, SR, & SA provided editorial input. All authors read and approved the final manuscript.

## Sources of support in the form of grants

Australian Government – National Health and Medical Research Council. Grants No: 601034 and 601025
